# Characteristics and long‐term prognosis of patients with reduced, mid‐range, and preserved ejection fraction: A systemic review and meta‐analysis

**DOI:** 10.1002/clc.23754

**Published:** 2022-01-18

**Authors:** Min Liang, Bo Bian, Qing Yang

**Affiliations:** ^1^ Department of Cardiology Tianjin Medical University General Hospital Tianjin People's Republic of China

**Keywords:** heart failure with mid‐range ejection fraction, heart failure with preserved ejection fraction, heart failure with reduced ejection fraction, mortality

## Abstract

**Aims:**

Patients with heart failure (HF) have a poor prognosis and are categorized by ejection fraction. We performed a meta‐analysis to compare baseline characteristics and long‐term outcomes of patients with heart failure with reduced (HFrEF), mid‐range (HFmrEF), and preserved ejection fraction (HFpEF).

**Methods and Results:**

A total of 27 prospective studies were included. Patients with HFpEF were older and had a higher proportion of females, hypertension, diabetes, and insufficient neuroendocrine antagonist treatments, while patients with HFrEF and HFmrEF had a higher prevalence of coronary heart disease and chronic kidney disease. After more than 1‐year of follow‐up, all‐cause mortality was significantly lower in patients with HFmrEF 9388/25 042 (37.49%) than those with HFrEF 39 333/90 023 (43.69%) and HFpEF 24 828/52 492 (47.30%) (*p* < .001). Cardiovascular mortality was lowest in patients with HFpEF 1130/9904 (11.41%), highest in patients with HFrEF 3419/16 277 (21.07%) mainly coming from HF death and sudden cardiac death, and middle in patients with HFmrEF 699/5171 (13.52%) and the non‐cardiovascular mortality was on the contrary. Subgroup analysis showed that in high‐risk patients with atrial fibrillation, the all‐cause mortality of HFpEF was significantly higher than both HFrEF and HFmrEF (*p* < .001). HF hospitalization was lowest in patients with HFmrEF 1822/5285 (34.47%), highest in patients with HFrEF 12 607/28 590 (44.10%) and middle in patients with HFpEF 8686/22 763 (38.16%) and the composite of all‐cause mortality and HF hospitalization was also observed similar results.

**Conclusions:**

In summary, patients with HFmrEF had the lowest incidence of all‐cause mortality and HF hospitalization, while the highest all‐cause mortality and HF hospitalization rates were HFpEF and HFrEF patients, respectively.

## 1 INTRODUCTION

Heart failure (HF) is a global pandemic affecting approximately 64.3 million people worldwide;^1^ furthermore, the total number of patients living with HF is increasing.^2^ At the same time, the poor prognosis of HF patients is another important and serious healthcare issue worldwide. Indeed, several studies have suggested similar mortality in patients with HF with reduced ejection fraction (HFrEF) and preserved ejection fraction (HFpEF),^3^ whereas others have demonstrated HFpEF patients have a substantially better prognosis compared with patients with HFrEF.^4^ The large meta‐analysis Global Group in Chronic Heart Failure (MAGGIC) study, pooling data from 30 cohort studies, showed that patients with HFpEF were at a significantly lower risk of death compared to their HFrEF counterparts.^5^ However, this analysis included retrospective studies, which probably lead to higher mortality rates due to selection bias in trials that included patients with common serious comorbidities, and use left ventricular ejection fraction (LVEF) 40% as the cutoff value for HF classification (LVEF < 40% for HFrEF, LVEF ≥ 40% for HFpEF, respectively) ignoring of HF with mid‐range ejection fraction (HFmrEF), a novel category that was defined LVEF 40%–49% in the 2016 European Society of Cardiology heart failure guideline.^6^ HFmrEF is considered as a transition between the HFpEF and HFrEF, it is imperative to investigate the differences between HFmrEF patients and those in the other two HF groups in terms of prognosis. More importantly, we need a better understanding of the causes of death in HF patients, which may contribute to better insights into the underlying pathophysiologic mechanisms and new treatments for improving patient outcomes.

Therefore, we conducted a meta‐analysis of prospective studies to compare clinical characteristics, assess the long‐term prognosis through all‐cause mortality and HF hospitalization of more than 1‐year follow‐up, and investigate the prevalence of cardiac/noncardiac causes of death among three categories of patients with HF.

## 2 METHODS

### 2.1 Ethics statement

As this study is a meta‐analysis, ethical approval was not required.

### 2.2 Search strategy

We performed a literature search in PubMed and Embase from the date of inception to March 2021. The following search formula (heart failure with reduced ejection fraction OR HFrEF) AND (heart failure with preserved ejection fraction OR HFpEF) AND (all‐cause mortality OR all‐cause death OR mortality OR death) was used in the English database. And language was restricted to English.

### 2.3 Study selection

Two independent reviewers screened the titles and abstracts of all selected articles. Only studies that were clearly irrelevant were excluded from this page. Any disagreements between the investigators were resolved by a third reviewer. Studies were included if they met the following criteria: (1) prospective studies; (2) providing numbers of events for all‐cause mortality in patients among three categories HF; (3) follow‐up period not less than 1 year. The definition of HF was made mainly based on 2016 ESC guideline,^6^ categorizing HF as LVEF ≥ 50%, 40%–49%, <40% as HFpEF, HFmrEF, and HFrEF, respectively, or the American College of Cardiology and American Heart Association guideline,^7^ which recommended LVEF ≥ 50%, 41%–49%, ≤40% as HFpEF, HFmrEF, and HFrEF, respectively. We excluded all retrospective studies or studies with unclear type, studies with a follow‐up period shorter than 1 year, and studies with insufficiently reported data.

### 2.4 Data extraction

Data were extracted by two independent reviewers. The extracted data included demographic features and key baseline clinical variables reported as means or medians with standard deviations (SD) or ranges from each study. We extracted absolute numbers for all‐cause and cardiovascular/non‐cardiovascular mortality and HF hospitalization. In addition, data on specific causes of cardiovascular mortality was also extracted. Disagreements were adjudicated by a third reviewer.

### 2.5 Statistical analysis

All statistical analyses were conducted by using Review Manager Version 5.4. The reported numbers of all‐cause and cardiovascular/non‐cardiovascular mortality and HF hospitalization in eligible studies were pooled for three categories of HF, followed by an estimation of an odds ratio (OR) with a 95% confidence interval (95% CI). The *Q* statistic was calculated and heterogeneity was quantified using the I^2^ statistic. Despite the significant heterogeneity between studies, we used a fix‐effects model to maintain the real sizes of the larger studies but beside that presented the results of a random‐effects methods wherever reasonable. A funnel plot was conducted to evaluate publication bias. We also conducted several subgroup analyses based on high‐risk patients, including acute HF, atrial fibrillation (AF), diabetes mellitus.

## 3 RESULTS

### 3.1 Search results

**Figure 1:**
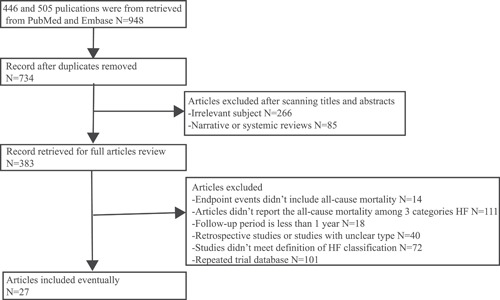
Flow chart of the search process result

The flow chart of the search strategy is provided (Figure 1). The search strategy retrieved a total of 948 studies from PubMed (446) and Embase (505), with 214 duplicated studies, and the remaining 734 studies were performed for titles and abstracts screening, among which 266 irrelevant subjects and 85 narrative or systemic reviews were excluded. Ultimately, 383 relevant articles were reviewed in full text. A further 355 articles were excluded after careful review of full text, including 14 articles without all‐cause mortality for endpoint events, 111 articles that did not report the all‐cause mortality among three categories of HF patients, 18 articles with a follow‐up period of less than 1 year, 40 articles for retrospective studies or studies with unclear type, 72 articles that did not meet the definition of HF classification and 101 articles for the repeated trial database. Consequently, 27 studies^8^, ^9^, ^10^, ^11^, ^12^, ^13^, ^14^, ^15^, ^16^, ^17^, ^18^, ^19^, ^20^, ^21^, ^22^, ^23^, ^24^, ^25^, ^26^, ^27^, ^28^, ^29^, ^30^, ^31^, ^32^, ^33^, ^34^ with a total of 167 557 patients met inclusion criteria and were included in the meta‐analysis.

### 3.2 Characteristics of included studies

The main characteristics of the included studies are summarized in Table 1. Among the included studies, only two were randomized controlled studies,^9^, ^27^ and the others were observational studies. The follow‐up duration varied from 1 to 6.3 years. In the included studies, 14 were from Asia, 9 from Europe, and 4 from North America. There were statistically differences in regard to baseline characteristics comparisons among three HF categories (Table 2). The baseline characteristics were as follows: age: 66.4 ± 12.5 versus 68.4 ± 12.9 versus 70.7 ± 12.8 years; male gender: 68.73% versus 61.48% versus 42.88%; coronary artery disease or ischemic HF: 55.41% versus 55.09% versus 42.13%; hypertension: 57.85% versus 65.11% versus 75.52%; diabetes: 32.24% versus 31.73% versus 34.72%; AF: 39.25% versus 47.50% versus 43.89%; chronic kidney disease: 23.09% versus 23.46% versus 20.47% among patients with HFrEF, HFmrEF, and HFpEF, respectively. Patients with HFpEF were significantly older than those with HFrEF and HFmrEF. The proportion of males and prevalence of coronary artery disease or ischemic HF and chronic kidney disease among HFpEF were significantly lower than those among HFrEF and HFmrEF, but hypertension and diabetes were more frequent in patients with HFpEF. The incidence of AF in patients with HFmrEF and HFpEF was significantly higher than that in patients with HFrEF. Drug applications, including ACEI or ARB, β‐blocker, aldosterone antagonists, and loop diuretics were the most used in HF patients with HFrEF, followed by HFmrEF, and the lowest application rate is HFpEF.

**Table 1:** Study characteristics

Study	Inclusion criteria	Country	Patients number (HFrEF/HFmrEF/HFpEF)	Outcomes (HFrEF/HFmrEF/HFpEF)	Follow up
Kawahira (2021)	Hospitalized patients with acute decompensated HF	Japan	164/104/198	ACM: 49/34/60	2.8 ± 1.5 years
SELFIE‐TR registry (2020)	Acute or chronic HF patients	Turkey	780/170/72	ACM: 155/31/17	1 year
Xu (2020)	Inpatients with HF	China	202/94/109	ACM: 21/8/2, HF hospitalization: 62/18/16, composite of ACM and HF hospitalization: 73/25/17	1 year
EXCEL trial (2020)	Hospitalized HF patients with left main coronary artery disease undergoing PCI or CABG	USA	74/152/1578	ACM: 13/14/96, CV mortality: 10/8/52	3 years
Song (2020)	Hospitalized HF patients	China	215/80/110	ACM: 36/8/13, HF hospitalization: 48/15/19, composite of ACM and HF hospitalization: 84/23/32	Median: 12 months (IQR: 6–20 months)
KorAHF registry (2020)	Hospitalized patients with acute HF	South Korea	3182/875/1357	ACM: 1609/472/726, CV mortality: 530/115/161, composite of ACM and HF readmission: 2532/703/1088	Median: 4.03 years (IQR: 1.39–5.58 years)
OPTIMIZE‐HF (2020)	Hospitalized HF patients	USA	3688/NA/1848	ACM: 2817/NA/1403, HF readmission: 2310/NA/959	Median: 2 years
ASIAN‐HF registry (2020)	Inpatients and outpatient with symptomatic HF	3 Asian regions	4737/NA/1114	ACM: 500/NA/60, CV mortality: 440/NA/46, non‐CV mortality: 170/NA/14	1 year
KCHF registry (2020)	Hospitalized patients with acute decompensated HF	Japan	1383/703/1631	ACM: 298/158/392, CV death: 203/97/223, (HF death: 128/65/131, SCD: 44/14/40, vascular death: 4/2/7, acute coronary syndrome: 5/0/4, stroke or intracranial hemorrhage: 8/9/21, other CV cause: 14/7/20), non‐CV death: 94/61/167, unknown death: 1/0/2	Median: 470 days (IQR: 357–649 days)
Kanagala (2020)	HF patients	United Kingdom	46/NA/140	ACM: 6/NA/22	Median: 1446 days (IQR: 1243–1613 days)
Gulf CARE registry (2020)	Hospitalized patients with acute HF	Seven Middle Eastern countries	2683/962/932	ACM: 548/152/181	1 year
Yee (2019)	Inpatients and outpatients with HF	USA	516/NA/151	ACM: 101/NA/13	16.6 ± 6.7 months
Vicent (2019)	Hospitalized patients with acute HF	Spain	583/227/610	ACM: 117/55/118, composite of ACM and HF readmission: 253/109/255	1 year
Vergaro (2019)	Chronic HF patients from the outpatient clinic	Italy	1539/623/629	ACM: 631/166/144, cardiac mortality: 415/74/54 (HF death: 277/40/37, SCD: 59/11/6, AMI: 27/10/7)	Median: 39 months (IQR: 17–79 months)
WET‐HF registry (2019)	Hospitalized patients with acute decompensated HF	Japan	1143/532/1277	ACM: 271/123/287, cardiac deaths: 69/45/128	Median: 724 days
Lin (2019)	Hospitalized patients with HF	Taiwan	158/NA/108	ACM: 27/NA/15	18 months
Norwegian HF registry (2019)	Ambulatory patients with stable chronic HF	Norway	7080/2086/1146	ACM: 3836/957/504	Median: 66 months (IQR: 33–105 months)
KorHF registry (2019)	Hospitalized patients due to HF	South Korea	1684/NA/727	ACM: 467/NA/226, composite of ACM and HF readmission: 729/NA/344	Median: 1121 days (IQR: 355–1887 days)
ESC‐HF‐LT registry (2018)	Outpatients with chronic HF and inpatients admitted for acute HF	21 European and Mediterranean countries	7476/2913/3672	ACM: 1240/403/548	800 days to 2.2 years
CHARM study (2018)	Patients with symptomatic HF	Sweden	4323/1322/1953	ACM: 1296/209/325, CV death: 1079/167/214, HF hospitalization: 1115/216/343	Median: 2.9 years
Lam (2018)	Patient in the hospital for primary diagnosis of HF or in the outpatient setting within 6 months of an episode of decompensated HF	New Zealand and Singapore	1209/256/574	ACM: 233/30/80, composite of ACM and HF hospitalization: 522/103/199	2 years
CHART‐2 study (2018)	Chronic HF patients from patient clinics or just before discharge	Japan	742/666/2893	ACM: 330/330/887	Median: 6.3 years
Gu (2018)	Hospitalized patients with a clinical diagnosis of HF and T2DM	China	481/131//290	ACM: 160/35/75, composite endpoints of ACM and HF hospitalization: 311/73/161	42 months
GWTG‐HF program (2017)	Hospitalized patients with acute HF	USA	18398/3285/18299	ACM: 13 847/2487/13 843, HF readmission: 8505/1416/7072	5 years
SwedeHF registry (2017)	HF patients from outpatient visits or hospital discharge	Sweden	22954/8897/9595	ACM: 8926/3367/4169, Composite of ACM or HF hospitalization: 13006/4551/5375	1 year
Pascual‐Figal (2017)	Ambulatory patients with chronic HF	Spain	2351/460/635	ACM: 776/128/178, CV death: 621/93/110, (HF death: 386/54/70, SCD: 190/29//24, other CV death: 45/10/16), non‐CV death: 155/35/68	4 years
Farre (2017)	Ambulatory HF patients	Spain	2232/504/844	ACM: 1023/221/444, CV death: 492/100/188, (HF death: 269/58/131, SCD: 101/13/12, other CV death: 122/29/45), non‐CV death: 265/72/163, unknown death: 266/49/93, HF hospitalization: 724/157/378, composite of ACM and HF hospitalization: 1277/272/564	Median: 3.36 years (IQR: 1.69–6.04 years)

Abbreviations: AMI, acute myocardial infarction; CABG, coronary artery bypass grafting; CV, cardiovascular; HF, heart failure; HFrEF, heart failure with reduced ejection fraction; HFmrEF, heart failure with mid‐range ejection fraction; HFpEF, heart failure with preserved ejection fraction; IQR, interquartile range; NA, not available; PCI, percutaneous coronary intervention; SCD, sudden cardiac death; T2DM, type 2 diabetes mellitus.

**Table 2:** Comparison of baseline characteristics among three categories of HF

		Values shown as weighted means ± SD or numbers (%)			*p* values		
Characteristics	Numbers of studies	HFrEF	HFmrEF	HFpEF	HFrEF versus HFpEF	HFrEF versus HFmrEF	HFmrEF versus HFpEF
*Demographic and clinical characteristics*							
Age	13	66.4 ± 12.5	68.4 ± 12.9	70.7 ± 12.8	<.001	<.001	<.001
Male gender	17	46 125/67 112 (68.73)	12 582/20 465 (61.48)	18 202/42 451 (42.88)	<.001	<.001	<.001
Coronary artery disease or ischemic HF	16	37 145/67 038 (55.41)	11 190/20 313 (55.09)	17 218/40 873 (42.13)	<.001	.83	<.001
Hypertension	17	38 826/67 112 (57.85)	13 325/20 465 (65.11)	32 059/42 451 (75.52)	<.001	<.001	<.001
Diabetes	16	21 482/66 631 (32.24)	6452/20 334 (31.73)	14 638/42 161 (34.72)	.71	.07	.01
Atrial fibrillation	15	23 533/59 958 (39.25)	8657/18 227 (47.50)	17 436/39 727 (43.89)	<.001	<.001	.2
Chronic kidney disease	7	7649/33 121 (23.09)	2105/8972 (23.46)	5722/27 951 (20.47)	<.001	.14	<.001
*Medications used*							
ACEI or ARB	16	54 080/66 897 (80.84)	15 154/20 385 (74.34)	25 925/42 341 (61.23)	<.001	<.001	<.001
Beta‐blocker	16	55 755/66 897 (83.34)	16 190/20 385 (79.42)	29 015/42 341 (68.53)	<.001	<.001	<.001
Aldosterone antagnoists	15	22 846/66 823 (34.19)	5415/20 233 (26.76)	8009/40 763 (19.65)	<.001	<.001	<.001
Loop diuretics	14	46 772/64 931 (72.03)	12 888/19 455 (66.25)	23 589/40 100 (58.83)	<.001	<.001	<.001

Abbreviations: ACEI, angiotensin enzyme inhibitor; ARB, angiotensin receptor blocker; HF, heart failure; HFrEF, heart failure with reduced ejection fraction; HFmrEF, heart failure with mid‐range ejection fraction; HFpEF, heart failure with preserved ejection fraction; SD, standard deviation.

### 3.3 Publication bias

Funnel plots were drawn for assessment of meta‐analysis in regard to all‐cause mortality among studies examining HFrEF versus HFpEF (Figure S1A), HFrEF versus HFmrEF (Figure S1B), and HFmrEF versus HFpEF (Figure S1C). The funnel plots for both groups of studies (HFrEF vs. HFpEF) look asymmetrical as there appear to be more studies missing on the left‐hand side and were relatively symmetrical between the studies of HFrEF versus HFmrEF and between HFmrEF versus HFpEF. The source of risk of bias across studies can only be speculated and could be attributed to publication bias, substantial heterogeneity, or even chance.

### 3.4 Study outcomes

#### 3.4.1 All‐cause mortality

**Figure 2:**
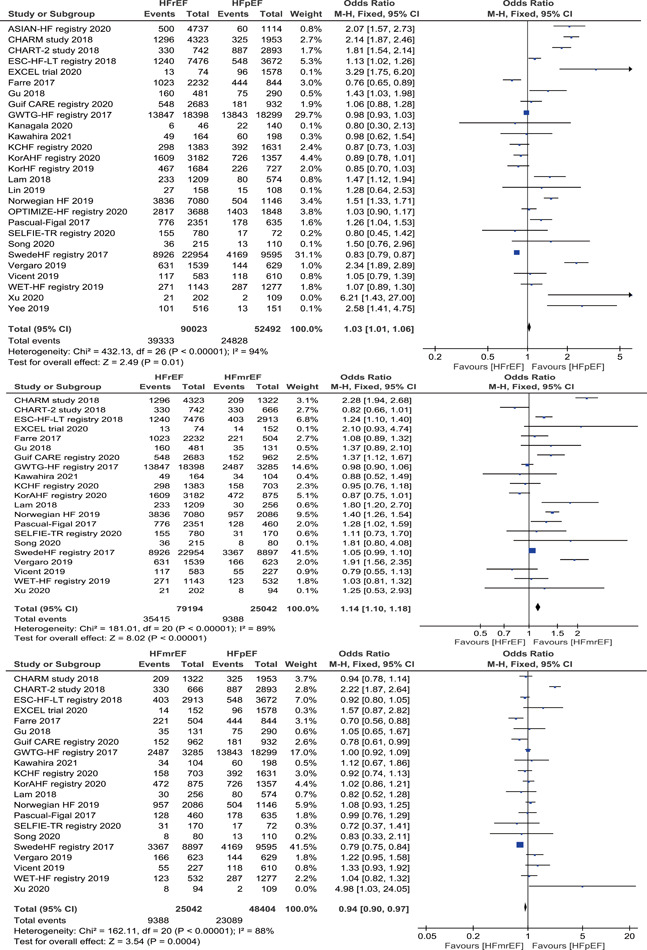
Forest plot of the odds ratio (OR) and 95% confidence interval (CI) for all‐cause mortality among three categories of HF. HF, heart failure; HFrEF, heart failure with reduced ejection fraction; HFmrEF, heart failure with mid‐range ejection fraction; HFpEF, heart failure with preserved ejection fraction

Patients with HFmrEF had lower all‐cause mortality 9388/25 042 (37.49%) than those with HFrEF 39 333/90 023 (43.69%) and HFpEF 24 828/52 492 (47.30%). Pooled data of 21 studies using the fixed‐effects model showed that the risk of all‐cause mortality was significantly lower in patients with HFmrEF than in those with HFrEF (OR = 1.14, 95% CI: 1.10–1.18, *p *< .001) and HFpEF (OR = 0.94, 95% CI: 0.90–0.97, *p *< .001), and Pooled data of 27 studies indicated that patients with HFrEF had lower all‐cause mortality compared with those with HFpEF (OR = 1.03, 95% CI: 1.01–1.06, *p* = .01) (Figure 2). There was significant heterogeneity between the included studies (*p* < .001 and i^2^ > 50%). Running the analysis using the random‐effects model showed that the risk of all‐cause mortality was still significantly lower in patients with HFmrEF than in those with HFrEF (OR = 1.2, 95% CI: 1.07–1.36, *p* = .002), but not significant when compared with those with HFpEF (OR = 1.03, 95% CI: 0.90–1.17, *p* = .7).

#### 3.4.2 Causes of death

**Figure 3:**
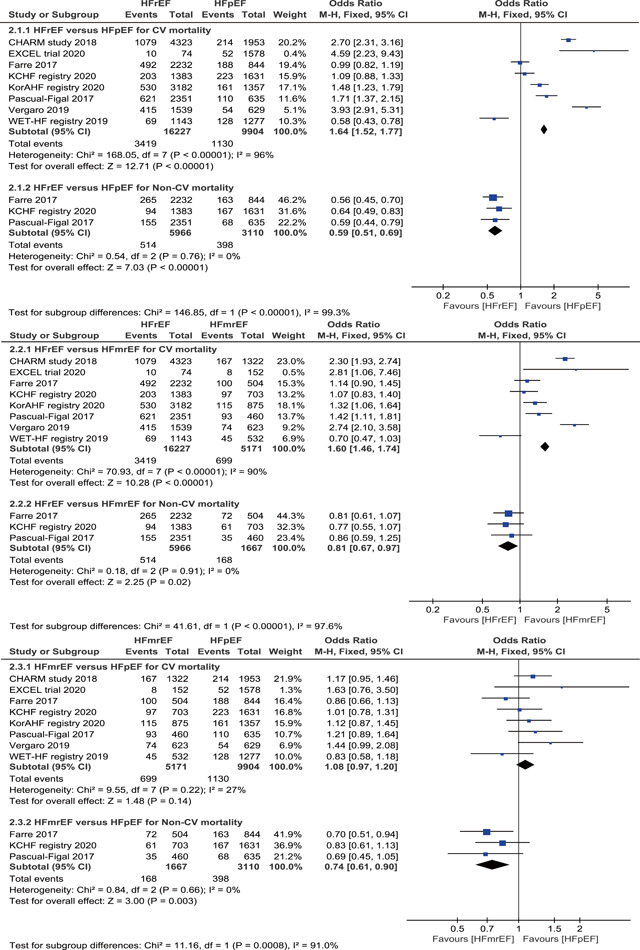
Forest plot of the odds ratio (OR) and 95% confidence interval (CI) for causes of death among three categories of HF. HF, heart failure; HFrEF, heart failure with reduced ejection fraction; HFmrEF, heart failure with mid‐range ejection fraction; HFpEF, heart failure with preserved ejection fraction

Eight studies provide data for cardiovascular mortality, which revealed that patients with HFrEF had higher cardiovascular mortality 3419/16 277 (21.07%) than those with HFmrEF 699/5171 (13.52%) and HFpEF 1130/9904 (11.41%), and meta‐analysis using the fixed‐effects model demonstrated a significantly higher risk of cardiovascular mortality in patients with HFrEF than in those with HFmrEF (OR = 1.60, 95% CI 1.46–1.74, *p* < .001) and HFpEF (OR = 1.64, 95% CI: 1.52–1.77, *p *< .001). In addition, a meta‐analysis from three studies indicated that patients with HFpEF had significantly higher non‐cardiovascular mortality 398/3110 (12.80%) than those with HFrEF 514/5966 (8.62%) and HFmrEF 168/1667 (10.08%) (Figure 3). Furthermore, we also analysis the cardiovascular‐specific death from four studies data, which displayed that patients with HFrEF were at significant higher risk of HF death 1060/7505 (14.12%) than those with HFmrEF 217/2290 (9.48%) and HFpEF 369/3739 (9.87%), and sudden cardiac death (SCD) were also significantly higher in patients with HFrEF 394/7505 (5.25%) than in those with HFmrEF 67/2290 (2.93%) and HFpEF 82/3739 (2.19%), but not significantly different between HFmrEF and HFpEF in regard to HF death and SCD (Figure S2). HF death accounted for 38.86%, 32.24%, 31.87% and SCD accounted for 14.44%, 9.96%, 7.08% of the total deaths in the three groups of HFrEF, HFmrEF, and HFpEF, respectively.

#### 3.4.3 Subgroup analysis

The subgroup analysis was performed based on high‐risk patients with acute HF or AF or diabetes mellitus. Among high‐risk patients, the risk of all‐cause mortality was still lower in patients with HFmrEF than those with HFrEF and HFpEF, but a statistically significant difference was only observed in AF patients with HFrEF and HFmrEF compared with patients with HFpEF from three studies, and there was no statistically significant difference in patients with acute HF from eight studies or diabetes mellitus from two studies among three categories of HF patients, with low heterogeneity (Figure S3).

#### 3.4.4 Other endpoints

Six studies provided data for HF hospitalization and nine studies for the composite of all‐cause mortality and HF hospitalization. There were 12 607/28 590 (44.10%), 1822/5285 (34.47%), and 8686/22 763 (38.16%) hospitalizations among HFrEF, HFmrEF, and HFpEF patients, respectively. When data are pooled using the fixed‐effects model, the risk of HF hospitalization was significantly lower in patients with HFmrEF than those with HFrEF and HFpEF, and significant differences were also observed between HFrEF and HFpEF. Similarly, the risk of composite of all‐cause mortality and HF hospitalization was significantly lower in patients with HFmrEF than those with HFrEF and HFpEF, but not significantly different between HFrEF and HFpEF (Figure S4).

## 4 DISCUSSION

This meta‐analysis consisting of recently published studies with substantial numbers of patients demonstrated marked differences in key baseline characteristics and long‐term prognosis, including all‐cause mortality, cardiovascular/non‐cardiovascular mortality, HF hospitalization, and composite of all‐cause mortality and HF hospitalization, among three HF categories. Patients with HFrEF were more often male, more frequently suffered from coronary artery disease or ischemic HF, and more often received the recommended medications, such as renin‐angiotensin system inhibitors and beta‐blockers. Baseline co‐morbidities, such as hypertension and diabetes, were more frequent in patients with HFpEF but AF was more common in patients with HFmrEF. Patients with HFmrEF had the lowest risk of all‐cause mortality, HF hospitalization and composite of all‐cause mortality and HF hospitalization. On the contrary, the highest incidence of all‐cause mortality was in patients with HFpEF, and patients with HFrEF had the highest HF hospitalization and composite of all‐cause mortality and HF hospitalization. Regarding the causes of death, HFrEF had the highest cardiovascular‐specific death, especially HF death and SCD.

HFmrEF is often termed as an “intermediate” phenotype between HFrEF and HFpEF but our findings challenge this. Based on our results, we observed that HFmrEF distinctly resembled HFrEF in coronary artery disease or ischemic HF, diabetes, and chronic kidney disease and was similar to HFpEF in AF except for age, sex, and hypertension, which was mostly different from a meta‐analysis consisting of 12 retrospective or prospective studies published 2018 whose results supported that demographics and comorbid conditions of HFmrEF were largely intermediate between those of HFpEF and HFrEF.^35^ More importantly, we also noticed that patients with HFmrEF had the lowest risk of all‐cause mortality, HF hospitalization, and the composite of these two components, partially consistent with the other two meta‐analyses,^35^, ^36^ which proved similar results about the lowest all‐cause mortality in HFmrEF but different results with respect to the lowest risk of HF hospitalization in HFpEF. Why do we observe a favorable prognosis for patients with HFmrEF? The existing evidence suggests that HFmrEF is characterized by mixed pathophysiology and a recent expert consensus focuses more on the pathophysiological mechanisms of HF rather than LVEF.^37^ As a subset of patients with HFmrEF appears to have more intense neurohormonal activation, therapies that block the neurohormonal axes may work in these patients, resembling the effects seen in HFrEF. Some observational studies and post hoc analyses of randomized controlled trials suggest that patients with HFmrEF benefit from medications that target the neurohormonal axes, including ACEI or ARB, β‐blocker, and aldosterone antagonists. Data from the Sweden HF registry suggested that ACEIs/ARBs were associated with a reduced risk of death irrespective of the presence or absence of coronary artery disease.^38^ Another analysis of the CHARM data proved candesartan significantly reduced the primary composite outcome of cardiovascular death or first HF hospitalization compared to placebo in HFrEF and HFmrEF but not in HFpEF.^27^ In an individual‐level meta‐analysis of 11 trials, β‐blockers halved cardiovascular mortality in patients with HFmrEF in sinus rhythm, regardless of ischemic or nonischemic etiology, which was similar to those observed in HFrEF, and β‐blockers helped to increase LVEF regardless of rhythm (sinus or AF) in the HFmrEF group, with a more pronounced benefit when the etiology was ischemic.^39^ Data from the Swedish Heart Failure Registry indicated that the one‐year mortality benefit of β‐blockers in patients with HFmrEF was restricted to those with underlying coronary artery disease.^38^ In our meta‐analysis, the characteristics of patients with HFmrEF, including comorbidities, such as coronary artery disease, diabetes, chronic kidney disease, and the medications they received were mostly similar to those of patients with HFrEF. From these results, treating HFmrEF with an evidence‐based therapy for HFrEF seems promising, and further studies should concentrate on this specific population with respect to the potential benefits of guideline‐directed medical therapy.

Of note, studies have shown that a considerable number of patients with HFmrEF transition to either HFrEF or HFpEF while on treatment, as do HFrEF and HFpEF. Among the included studies, only one study by Farre^34^ provided changes in LVEF of patients with alive at 1 year, which shown that 62% of HFmrEF patients still remained LVEF 40~50% and 24% and 33% of HFmrEF patients transitioned to HFrEF and HFpEF, respectively, and there were no differences in mortality between patients who remained in HFmrEF group and those who changed to HFrEF, while survival was significantly higher in those patients who evolved to the HFpEF group. Unfortunately, other included studies failed to provide more information about this. A prospective cohort of 1821 chronic HF patients demonstrated that HF‐recovered patients, defined as LVEF enrollment ≥50% but prior LVEF < 50%, had the best prognosis in terms of death, cardiac transplantation, and ventricular assist device placement than HFrEF (LVEF < 50%) and HFpEF (LVEF always ≥ 50%) patients.^40^ These suggest that HF‐recovered population may represent a distinct HF phenotype and we need to further investigate pathophysiological differences in these patient populations in an effort to better tailor therapy.

Unexpectedly, the highest risk of all‐cause mortality is in HFpEF patients, rather than HFrEF patients, which may be explained by a high proportion of higher age and females and the association of the markedly higher burden of co‐comorbidities, such as hypertension, diabetes, and AF, and our subgroup analysis confirmed the highest all‐cause mortality risk of HFpEF in the high‐risk population of AF. A multinational prospective observational study aimed at characterizing HFpEF (LVEF ≥ 45%) also confirmed that HFpEF was associated with higher age, female gender, hypertension, AF/flutter, and numerous non‐cardiovascular co‐morbidities, such as anemia, renal dysfunction, diabetes, lung disease, and cancer and the prognosis was determined by non‐cardiovascular co‐morbidities.^41^ More critically, patients with HFpEF received application of renin–angiotensin system blockers and β‐blockers significantly less than those with HFrEF and HFmrEF from our results. Because the findings of randomized trials of neurohormonal modulation have been neutral in HFpEF and consistently positive in HFrEF, which results in the infrequent use of neuroendocrine antagonists in HFpEF. A recently published meta‐analysis consisting of randomized controlled trials involving patients with HFpEF revealed that β‐blockers, ACEI, ARB, and mineralocorticoid receptor antagonists treatment has little or no effect on all‐cause mortality, and β‐blockers maybe have a possible reduction in cardiovascular mortality, mineralocorticoid receptor antagonists probably reduces HF hospitalization, and other drugs have no observed benefits for cardiovascular mortality and heart hospitalization.^42^ The PARAGON‐HF trial, including 4822 patients with HFpEF of LVEF ≥ 45%, demonstrated that sacubitril‐valsartan, a drug currently used to replace ACEI/ARB in the treatment of HFrEF, did not significantly lower the rate of total hospitalizations for HF, and death from cardiovascular causes compared with valsartan and sub‐group analysis identified lower risk reduction for the primary outcome among those with LVEF no more than 57%.^43^ Thus, guidelines offer no specific treatment recommendations regarding the use of these therapies in HFpEF beyond the management of comorbidities. Furthermore, regarding the cause of death, our study indicated that the non‐cardiovascular deaths of patients with HFpEF were significantly higher than those with HFrEF and HFmrEF. In a KCHF study,^16^ infection was the leading cause of non‐cardiovascular death, then followed by a malignant tumor. Regretfully, however, our results cannot add further information on non‐cardiovascular death causes of patients with HFpEF due to the lack of statistical power. Taken together, we should not only seek effective methods to treat HFpEF itself to improve prognosis but also pay more attention to the management of comorbidities.

HFrEF is the most commonly studied subgroup of HF and there are treatments proved to be effective in this phenotype, including ACEIs/ARBs or angiotensin receptor neprilysin inhibitor (ARNI) recently, β‐blockers, and aldosterone antagonists, which are definitely recommended as evidence‐based treatments by the ESC^6^ and American College of Cardiology/American Heart Association (ACC/AHA)^44^ yielding a reduction in mortality and morbidity, which are also confirmed in this article. The evidence‐based treatments were significantly higher in HFrEF patients than both HFmrEF and HFpEF patients, which may explain why the all‐cause mortality of patients with HFrEF was lower than those of patients with HFpEF, rather than the highest, in spite of the high prevalence of coronary artery disease or ischemic HF, which is one of the major contributing causes of death in HF populations. Hence, these drugs should be initiated as soon as possible, and they should be titrated up to the highest dose according to patient tolerability. Moreover, the cardiovascular mortality in patients with HFrEF was significantly higher than those with HFmrEF and HFpEF, especially HF death and SCD.

In addition, we conducted subgroup analyses of high‐risk populations and found that there was no difference in all‐cause mortality among the three categories of patients with acute HF or type 2 diabetes except for AF. This result suggested no association between the LVEF strata and the prognosis in patients with acute HF, which was not consistent with previous observations in chronic HF.^45^ The differences may are attributed to dynamic LVEF changes as a result of correction of the underlying cardiac defect in the cases of hospitalization for acute HF, especially acute decompensated HF, and prognostic events occur during the vulnerable phase after hospital discharge, which is largely the results of insufficient treatments during the index hospitalization or nonadherence to the treatment associated with socioeconomic status or lack of education in this phase.^46^ Thus, simply trying to evaluate the long‐term event rate in patients with acute HF according to the LVEF strata may be both difficult and inappropriate. AF was more common in patients with HFmrEF and HFpEF, and AF was more strongly associated with all‐cause mortality in the HFpEF group than in the HFrEF and HFmrEF group in our meta‐analysis, which was contrary to the result of a previous meta‐analysis in favor of significantly higher all‐cause mortality in AF patients with HFrEF compared with HFpEF.^47^ However, A retrospective study supported that AF was associated with increased all‐cause mortality in patients with HFpEF but not in patients with HFrEF.^48^ Furthermore, a recently published meta‐analysis evaluating the relationship between AF and mortality risk in HFpEF, showed that AF was associated with an 11% increased risk of all‐cause mortality in patients with HFpEF and AF was an independent predictor of HF hospitalization, cardiovascular death, and stroke.^49^ Future studies should focus on the underlying mechanisms of these dual conditions and seek potential therapeutic strategies.

This meta‐analysis had several limitations. First, the populations of included studies were heterogeneous concerning the baseline characteristics and the size of the prevalence of comorbidities. Another source of heterogeneity is due to the different sizes of included studies, ranging from a few hundred to tens of thousands of samples. Thus, running the mortality and hospitalization analyses in the fixed‐effects model was more realistic. Second, some inculuded studies did not provide sufficient data for analyses regarding baseline characteristics and other endpoints, including cardiovascular/non‐cardiovascular mortality, HF hospitalization, and combination of all‐cause mortality and HF hospitalization, resulting in lacked statistical power. This article only took available key baseline characteristics into consideration and did not include body mass index, chronic kidney disease, chronic obstructive pulmonary disease, anemia, or HF‐related echocardiographic parameters other than LVEF in the analyses. Finally, the HFrEF population constituted almost of the whole analyzed population, while the HFmrEF and HFpEF population accounted for a small proportion, which may be attributed to imbalanced recruitment and registration. Thus, compared with well‐treated populations in randomized controlled trials, the all‐cause mortality estimates may be higher and a time effect is possible. Accordingly, the results of this study should be interpreted cautiously.

## 5 CONCLUSIONS

In conclusion, the long‐term prognoses, including all‐cause mortality, HF hospitalization, and composite of all‐cause mortality and HF hospitalization, for patients with HFmrEF were significantly lower than those for patients with HFpEF and HFrEF. Patients with HFpEF were associated with a higher risk of all‐cause mortality, which also has been observed in patients at high risk of AF and non‐cardiovascular mortality. Patients with HFrEF were related to a higher risk of cardiovascular mortality, especially HF death and SCD, and HF hospitalization and composite of all‐cause mortality and HF hospitalization. These findings should encourage more research on patient characteristics, mortality, and the effect of HF therapies to improve outcomes of patients, especially for the management of comorbidities of HFpEF.

## Supporting information

Supporting information.

Supporting information.

Supporting information.

Supporting information.

## Data Availability

The data that support the findings of this study are available from the corresponding author upon reasonable request.
