# Supplementary material for: Characteristics and long‐term prognosis of patients with reduced, mid‐range, and preserved ejection fraction: A systemic review and meta‐analysis
Source: Clin Cardiol. 2022 Jan 18;45(1):5–17. doi: 10.1002/clc.23754 (PMC8799045; doi:10.1002/clc.23754)
Supplement: Supplementary file 1 — Supporting information. [file CLC-45-5-s001.pdf]

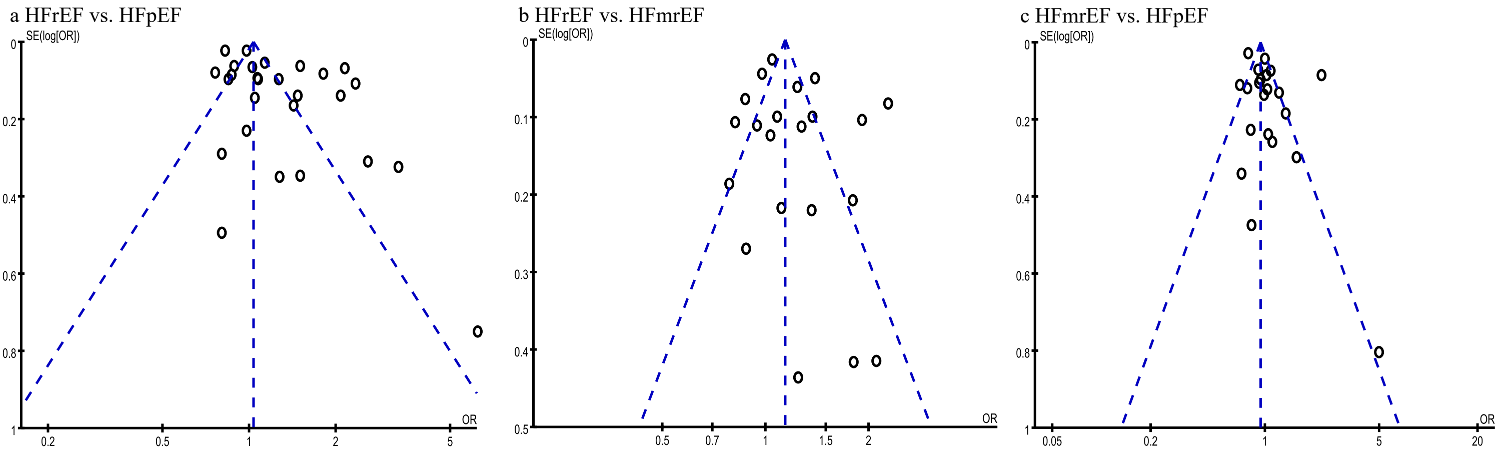

eFigure 1. Funnel plots of studies assessing all-cause mortality among three categories HF patients.
