# Supplementary material for: Characteristics and long‐term prognosis of patients with reduced, mid‐range, and preserved ejection fraction: A systemic review and meta‐analysis
Source: Clin Cardiol. 2022 Jan 18;45(1):5–17. doi: 10.1002/clc.23754 (PMC8799045; doi:10.1002/clc.23754)
Supplement: Supplementary file 2 — Supporting information. [file CLC-45-5-s004.pdf]

Study or Subgroup	HFrEF Events	HFrEF Total	HFpEF Events	HFpEF Total	Weight	Odds Ratio M-H, Fixed, 95% CI
**3.1.1 HFrEF versus HFpEF for HF death**						
Farre 2017	269	2232	131	844	40.6%	0.75 [0.60, 0.93]
KCHF registry 2020	128	1383	131	1631	26.5%	1.17 [0.91, 1.51]
Pascual-Figal 2017	386	2351	70	635	22.4%	1.59 [1.21, 2.08]
Vergaro 2019	277	1539	37	629	10.5%	3.51 [2.46, 5.02]
**Subtotal (95% CI)**		**7505**		**3739**	**100.0%**	**1.34 [1.17, 1.52]**
Total events	1060		369			
Heterogeneity: Chi² = 56.41, df = 3 (P < 0.00001); I² = 95%						
Test for overall effect: Z = 4.37 (P < 0.0001)						
**3.1.2 HFrEF versus HFpEF for SCD**						
Farre 2017	101	2232	12	844	17.5%	3.29 [1.80, 6.01]
KCHF registry 2020	44	1383	40	1631	37.4%	1.31 [0.85, 2.02]
Pascual-Figal 2017	190	2351	24	635	36.5%	2.24 [1.45, 3.45]
Vergaro 2019	59	1539	6	629	8.6%	4.14 [1.78, 9.64]
**Subtotal (95% CI)**		**7505**		**3739**	**100.0%**	**2.24 [1.74, 2.88]**
Total events	394		82			
Heterogeneity: Chi² = 9.48, df = 3 (P = 0.02); I² = 68%						
Test for overall effect: Z = 6.21 (P < 0.00001)						

Odds Ratio M-H, Fixed, 95% CI

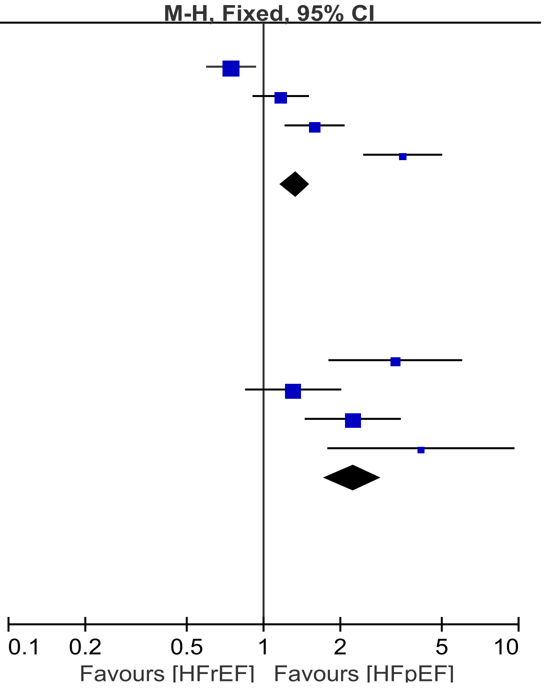

Study or Subgroup	HFrEF Events	HFrEF Total	HFmrEF Events	HFmrEF Total	Weight	Odds Ratio M-H, Fixed, 95% CI
**3.2.1 HFrEF versus HFmrEF for HF death**						
Farre 2017	269	2232	58	504	29.3%	1.05 [0.78, 1.42]
KCHF registry 2020	128	1383	65	703	27.6%	1.00 [0.73, 1.37]
Pascual-Figal 2017	386	2351	54	460	26.6%	1.48 [1.09, 2.00]
Vergaro 2019	277	1539	40	623	16.5%	3.20 [2.26, 4.52]
**Subtotal (95% CI)**		**7505**		**2290**	**100.0%**	**1.51 [1.29, 1.76]**
Total events	1060		217			
Heterogeneity: Chi² = 30.15, df = 3 (P < 0.00001); I² = 90%						
Test for overall effect: Z = 5.16 (P < 0.00001)						
**3.2.2 HFrEF versus HFmrEF for SCD**						
Farre 2017	101	2232	13	504	20.7%	1.79 [1.00, 3.22]
KCHF registry 2020	44	1383	14	703	18.4%	1.62 [0.88, 2.97]
Pascual-Figal 2017	190	2351	29	460	45.6%	1.31 [0.87, 1.96]
Vergaro 2019	59	1539	11	623	15.4%	2.22 [1.16, 4.25]
**Subtotal (95% CI)**		**7505**		**2290**	**100.0%**	**1.60 [1.23, 2.09]**
Total events	394		67			
Heterogeneity: Chi² = 2.08, df = 3 (P = 0.56); I² = 0%						
Test for overall effect: Z = 3.48 (P = 0.0005)						

Odds Ratio M-H, Fixed, 95% CI

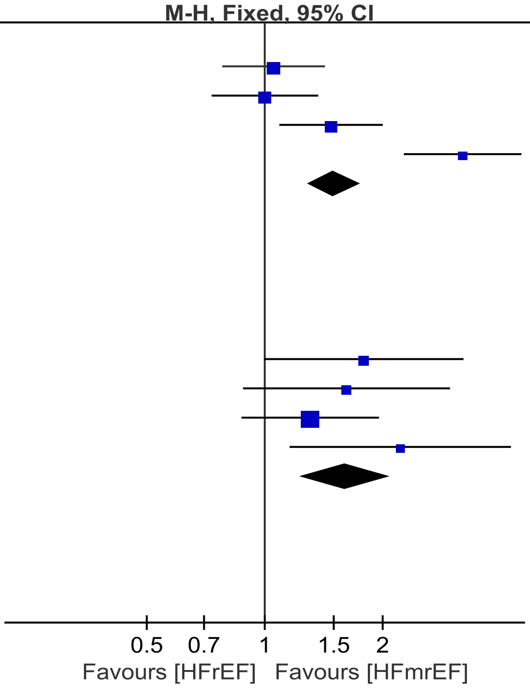

Study or Subgroup	HFmrEF Events	HFmrEF Total	HFpEF Events	HFpEF Total	Weight	Odds Ratio M-H, Fixed, 95% CI
**3.3.1 HFmrEF versus HFpEF for HF death**						
Farre 2017	58	504	131	844	35.4%	0.71 [0.51, 0.99]
KCHF registry 2020	65	703	131	1631	29.3%	1.17 [0.85, 1.59]
Pascual-Figal 2017	54	460	70	635	21.2%	1.07 [0.74, 1.57]
Vergaro 2019	40	623	37	629	14.1%	1.10 [0.69, 1.74]
**Subtotal (95% CI)**		**2290**		**3739**	**100.0%**	**0.97 [0.82, 1.17]**
Total events	217		369			
Heterogeneity: Chi² = 5.38, df = 3 (P = 0.15); I² = 44%						
Test for overall effect: Z = 0.28 (P = 0.78)						
**3.3.2 HFmrEF versus HFpEF for SCD**						
Farre 2017	13	504	12	844	15.3%	1.84 [0.83, 4.05]
KCHF registry 2020	14	703	40	1631	41.3%	0.81 [0.44, 1.50]
Pascual-Figal 2017	29	460	24	635	33.1%	1.71 [0.98, 2.98]
Vergaro 2019	11	623	6	629	10.3%	1.87 [0.69, 5.08]
**Subtotal (95% CI)**		**2290**		**3739**	**100.0%**	**1.37 [0.98, 1.92]**
Total events	67		82			
Heterogeneity: Chi² = 4.34, df = 3 (P = 0.23); I² = 31%						
Test for overall effect: Z = 1.86 (P = 0.06)						

Odds Ratio M-H, Fixed, 95% CI

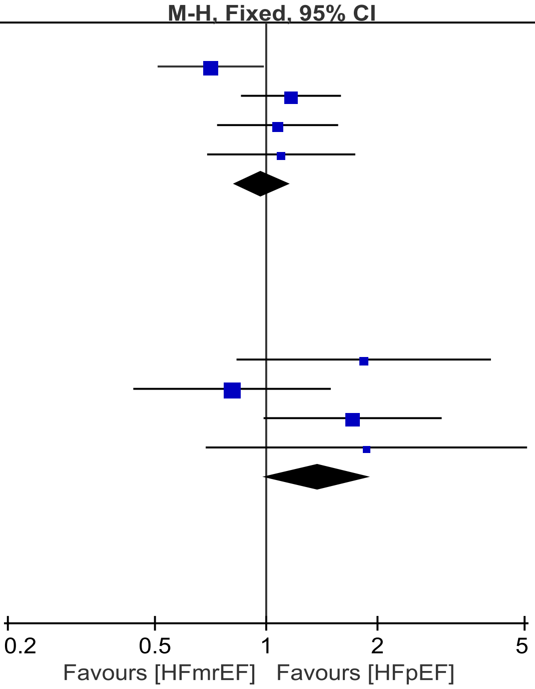

eFigure 2. Forest plot of the odds ratio (OR) and 95% confidence interval (CI) for causes of CV mortality among three categories HF.
