# Supplementary material for: Characteristics and long‐term prognosis of patients with reduced, mid‐range, and preserved ejection fraction: A systemic review and meta‐analysis
Source: Clin Cardiol. 2022 Jan 18;45(1):5–17. doi: 10.1002/clc.23754 (PMC8799045; doi:10.1002/clc.23754)
Supplement: Supplementary file 3 — Supporting information. [file CLC-45-5-s002.pdf]

Study or Subgroup	HFrEF Events	HFrEF Total	HFpEF Events	HFpEF Total	Weight	Odds Ratio M-H, Fixed, 95% CI
**4.1.1 in acute HF patients**						
Guif CARE registry 2020	548	2683	181	932	4.4%	1.06 [0.88, 1.28]
GWTG-HF registry 2017	13847	18398	13843	18299	70.9%	0.98 [0.93, 1.03]
Kawahira 2021	49	164	60	198	0.8%	0.98 [0.62, 1.54]
KCHF registry 2020	298	1383	392	1631	5.8%	0.87 [0.73, 1.03]
KorAHF registry 2020	1609	3182	726	1357	10.4%	0.89 [0.78, 1.01]
KorHF registry 2019	150	1142	61	528	1.5%	1.16 [0.84, 1.59]
Vicent 2019	117	583	118	610	1.9%	1.05 [0.79, 1.39]
WET-HF registry 2019	271	1143	287	1277	4.3%	1.07 [0.89, 1.30]
**Subtotal (95% CI)**		**28678**		**24832**	**100.0%**	**0.98 [0.94, 1.02]**
Total events	16889		15668			
Heterogeneity: Chi² = 7.00, df = 7 (P = 0.43); I² = 0%						
Test for overall effect: Z = 1.23 (P = 0.22)						
**4.1.2 in AF patients**						
ESC-HF-LT registry 2018	396	1946	296	1438	10.4%	0.99 [0.83, 1.17]
KorAHF registry 2020	442	921	338	614	8.1%	0.75 [0.61, 0.92]
SwedeHF registry 2017	5355	12187	2882	6250	81.6%	0.92 [0.86, 0.97]
**Subtotal (95% CI)**		**15054**		**8302**	**100.0%**	**0.91 [0.86, 0.96]**
Total events	6193		3516			
Heterogeneity: Chi² = 4.16, df = 2 (P = 0.12); I² = 52%						
Test for overall effect: Z = 3.33 (P = 0.0009)						
**4.1.3 in T2DM patients**						
Gu 2018	160	481	75	290	52.8%	1.43 [1.03, 1.98]
Guif CARE registry 2020	108	1268	42	475	47.2%	0.96 [0.66, 1.39]
**Subtotal (95% CI)**		**1749**		**765**	**100.0%**	**1.21 [0.94, 1.54]**
Total events	268		117			
Heterogeneity: Chi² = 2.49, df = 1 (P = 0.11); I² = 60%						
Test for overall effect: Z = 1.50 (P = 0.13)						

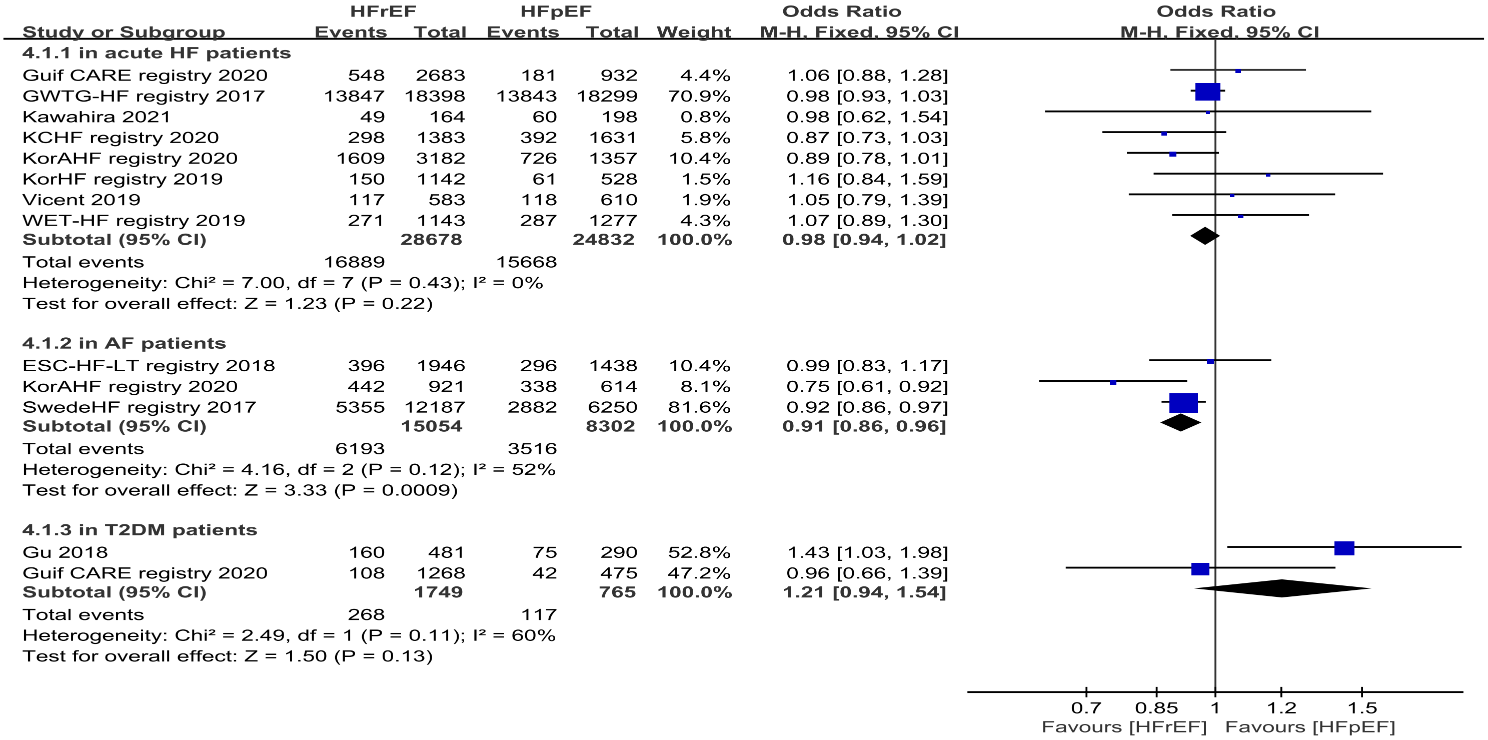

Study or Subgroup	HFrEF Events	HFrEF Total	HFmrEF Events	HFmrEF Total	Weight	Odds Ratio M-H, Fixed, 95% CI
**4.2.1 in acute HF patients**						
Guif CARE registry 2020	548	2683	152	962	9.0%	1.37 [1.12, 1.67]
GWTG-HF registry 2017	13847	18398	2487	3285	52.9%	0.98 [0.90, 1.06]
Kawahira 2021	49	164	34	104	1.5%	0.88 [0.52, 1.49]
KCHF registry 2020	298	1383	158	703	8.3%	0.95 [0.76, 1.18]
KorAHF registry 2020	1609	3182	472	875	18.6%	0.87 [0.75, 1.01]
Vicent 2019	117	583	55	227	3.2%	0.79 [0.55, 1.13]
WET-HF registry 2019	271	1143	123	532	6.5%	1.03 [0.81, 1.32]
**Subtotal (95% CI)**		**27536**		**6688**	**100.0%**	**0.99 [0.93, 1.05]**
Total events	16739		3481			
Heterogeneity: Chi² = 15.12, df = 6 (P = 0.02); I² = 60%						
Test for overall effect: Z = 0.43 (P = 0.66)						
**4.2.2 in AF patients**						
ESC-HF-LT registry 2018	396	1946	153	828	8.2%	1.13 [0.92, 1.39]
KorAHF registry 2020	442	921	189	348	6.8%	0.78 [0.61, 0.99]
SwedeHF registry 2017	5355	12187	2273	5312	85.0%	1.05 [0.98, 1.12]
**Subtotal (95% CI)**		**15054**		**6488**	**100.0%**	**1.04 [0.98, 1.10]**
Total events	6193		2615			
Heterogeneity: Chi² = 5.98, df = 2 (P = 0.05); I² = 67%						
Test for overall effect: Z = 1.15 (P = 0.25)						
**4.2.3 in T2DM patients**						
Gu 2018	160	481	35	131	37.5%	1.37 [0.89, 2.10]
Guif CARE registry 2020	108	1268	47	515	62.5%	0.93 [0.65, 1.33]
**Subtotal (95% CI)**		**1749**		**646**	**100.0%**	**1.09 [0.83, 1.44]**
Total events	268		82			
Heterogeneity: Chi² = 1.84, df = 1 (P = 0.17); I² = 46%						
Test for overall effect: Z = 0.63 (P = 0.53)						

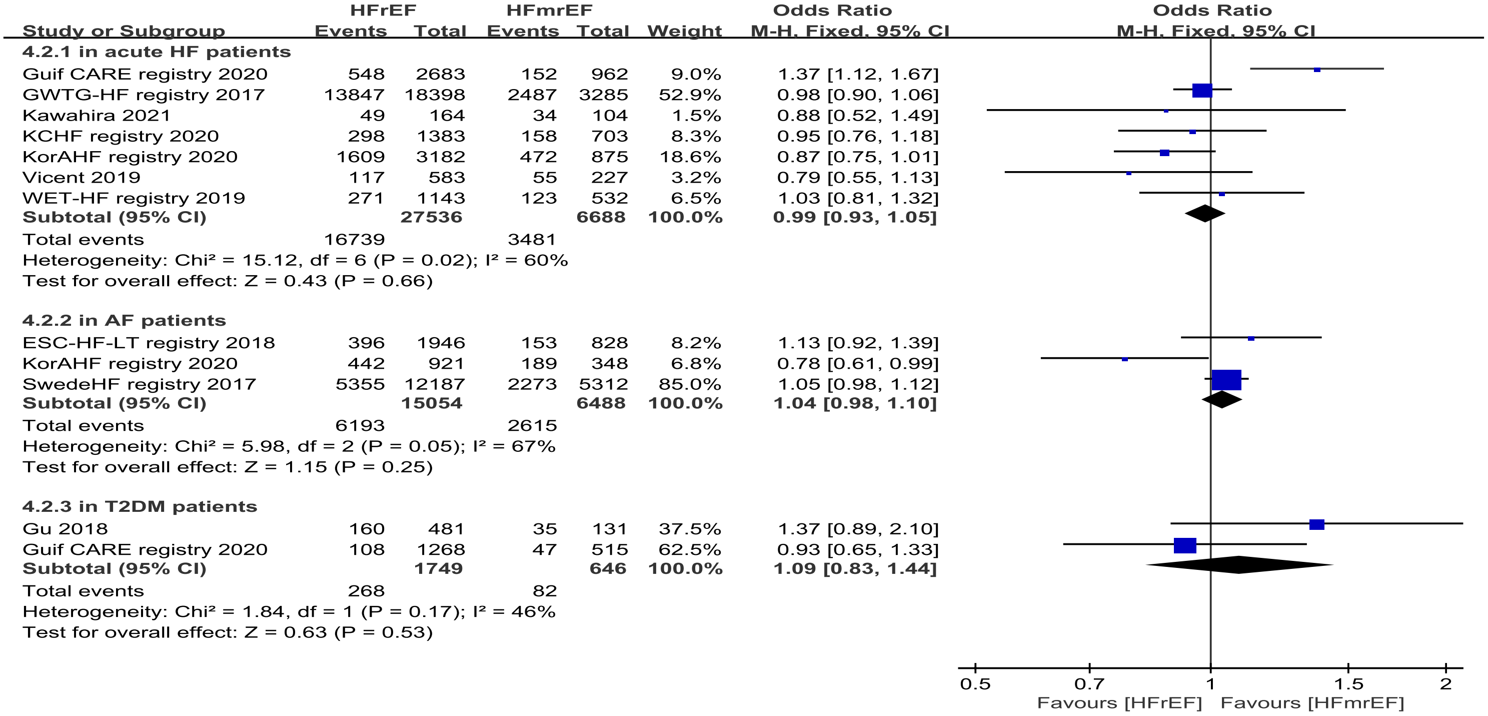

Study or Subgroup	HFmrEF Events	HFmrEF Total	HFpEF Events	HFpEF Total	Weight	Odds Ratio M-H, Fixed, 95% CI
**4.3.1 in acute HF patients**						
Guif CARE registry 2020	152	962	181	932	8.5%	0.78 [0.61, 0.99]
GWTG-HF registry 2017	2487	3285	13843	18299	55.9%	1.00 [0.92, 1.09]
Kawahira 2021	34	104	60	198	1.5%	1.12 [0.67, 1.86]
KCHF registry 2020	158	703	392	1631	10.0%	0.92 [0.74, 1.13]
KorAHF registry 2020	472	875	726	1357	14.3%	1.02 [0.86, 1.21]
Vicent 2019	55	227	118	610	2.7%	1.33 [0.93, 1.92]
WET-HF registry 2019	123	532	287	1277	7.1%	1.04 [0.82, 1.32]
**Subtotal (95% CI)**		**6688**		**24304**	**100.0%**	**0.99 [0.93, 1.06]**
Total events	3481		15607			
Heterogeneity: Chi² = 7.57, df = 6 (P = 0.27); I² = 21%						
Test for overall effect: Z = 0.29 (P = 0.77)						
**4.3.2 in AF patients**						
ESC-HF-LT registry 2018	153	828	296	1438	9.8%	0.87 [0.70, 1.09]
KorAHF registry 2020	189	348	338	614	6.2%	0.97 [0.75, 1.26]
SwedeHF registry 2017	2273	5312	2882	6250	84.0%	0.87 [0.81, 0.94]
**Subtotal (95% CI)**		**6488**		**8302**	**100.0%**	**0.88 [0.82, 0.94]**
Total events	2615		3516			
Heterogeneity: Chi² = 0.56, df = 2 (P = 0.75); I² = 0%						
Test for overall effect: Z = 3.71 (P = 0.0002)						
**4.3.3 in T2DM patients**						
Gu 2018	35	131	75	290	46.3%	1.05 [0.65, 1.67]
Guif CARE registry 2020	47	515	42	475	53.7%	1.04 [0.67, 1.60]
**Subtotal (95% CI)**		**646**		**765**	**100.0%**	**1.04 [0.76, 1.43]**
Total events	82		117			
Heterogeneity: Chi² = 0.00, df = 1 (P = 0.98); I² = 0%						
Test for overall effect: Z = 0.24 (P = 0.81)						

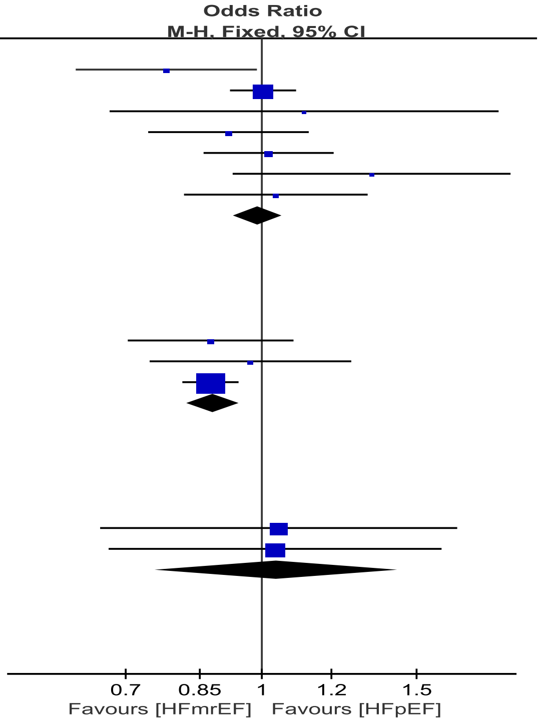

eFigure 3. Forest plot of the odds ratio (OR) and 95% confidence interval (CI) for all-cause mortality in different subgroups according to EF category.
