# Supplementary material for: Characteristics and long‐term prognosis of patients with reduced, mid‐range, and preserved ejection fraction: A systemic review and meta‐analysis
Source: Clin Cardiol. 2022 Jan 18;45(1):5–17. doi: 10.1002/clc.23754 (PMC8799045; doi:10.1002/clc.23754)
Supplement: Supplementary file 4 — Supporting information. [file CLC-45-5-s003.pdf]

Study or Subgroup	HFrEF Events	HFrEF Total	HFpEF Events	HFpEF Total	Weight	Odds Ratio M-H, Fixed, 95% CI
**6.1.1 HF hospitalization**						
CHARM study 2018	958	3855	242	1553	5.2%	1.79 [1.53, 2.09]
Farre 2017	724	2232	378	844	7.5%	0.59 [0.50, 0.70]
GWTG-HF registry 2017	8505	18398	7072	18299	77.0%	1.36 [1.31, 1.42]
OPTIMIZE-HF registry 2020	2310	3688	959	1848	9.6%	1.55 [1.39, 1.74]
Song 2020	48	215	19	110	0.4%	1.38 [0.76, 2.48]
Xu 2020	62	202	16	109	0.3%	2.57 [1.40, 4.73]
**Subtotal (95% CI)**		**28590**		**22763**	**100.0%**	**1.35 [1.30, 1.40]**
Total events	12607		8686			
Heterogeneity: Chi² = 122.74, df = 5 (P < 0.00001); I² = 96%						
Test for overall effect: Z = 16.12 (P < 0.00001)						
**6.1.2 Composite of ACM and HF hospitalization**						
Farre 2017	1277	2232	564	844	7.8%	0.66 [0.56, 0.78]
Gu 2018	311	481	161	290	1.6%	1.47 [1.09, 1.97]
KorAHF registry 2020	2532	3182	1088	1357	6.9%	0.96 [0.82, 1.13]
KorHF registry 2019	336	1142	138	528	3.0%	1.18 [0.93, 1.49]
Lam 2018	522	1209	199	574	3.4%	1.43 [1.17, 1.76]
Song 2020	84	215	32	110	0.6%	1.56 [0.95, 2.56]
SwedeHF registry 2017	13006	22954	5375	9595	73.2%	1.03 [0.98, 1.08]
Vicent 2019	253	583	255	610	3.1%	1.07 [0.85, 1.34]
Xu 2020	73	202	17	109	0.3%	3.06 [1.69, 5.53]
**Subtotal (95% CI)**		**32200**		**14017**	**100.0%**	**1.03 [0.99, 1.07]**
Total events	18394		7829			
Heterogeneity: Chi² = 59.95, df = 8 (P < 0.00001); I² = 87%						
Test for overall effect: Z = 1.40 (P = 0.16)						

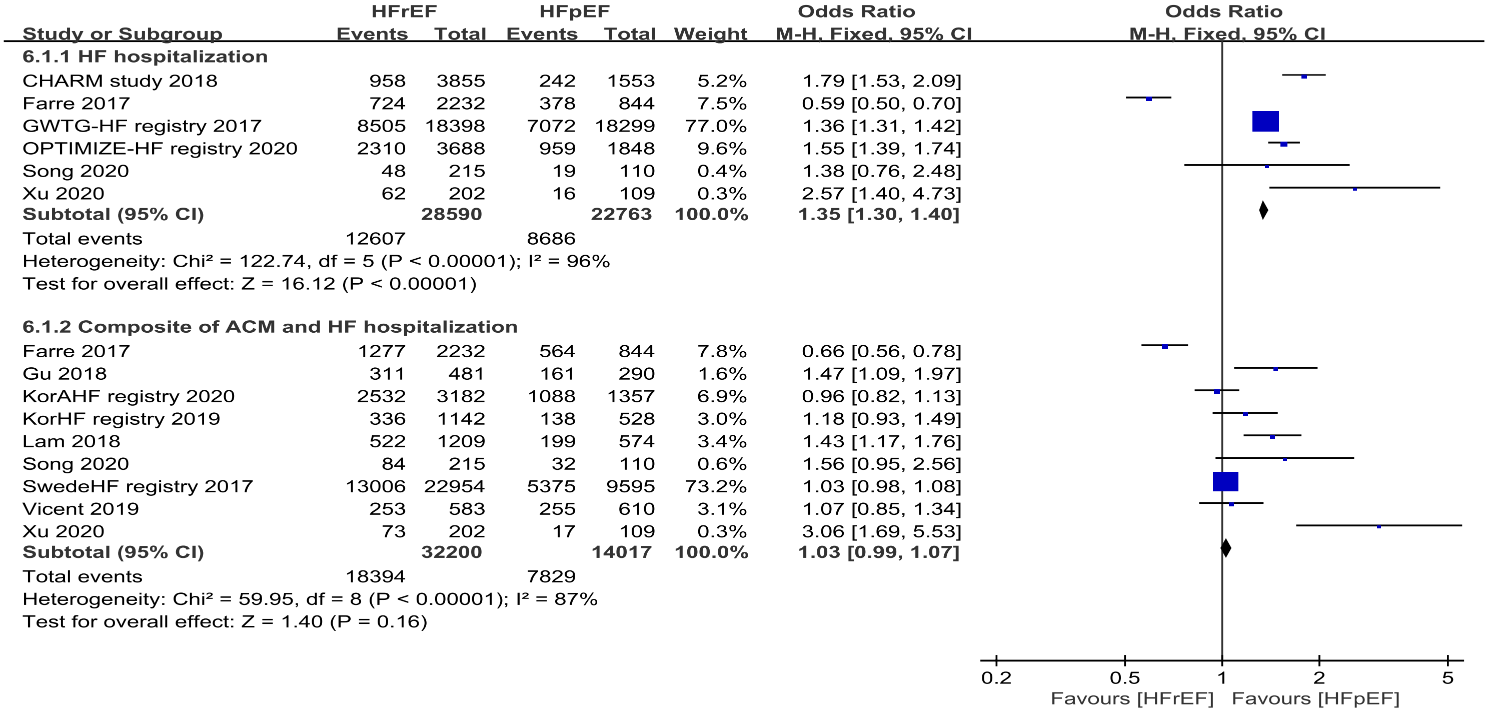

Study or Subgroup	HFrEF Events	HFrEF Total	HFmrEF Events	HFmrEF Total	Weight	Odds Ratio M-H, Fixed, 95% CI
**6.2.1 HF hospitalization**						
CHARM study 2018	1115	4323	216	1322	14.1%	1.78 [1.52, 2.09]
Farre 2017	724	2232	157	504	9.9%	1.06 [0.86, 1.31]
GWTG-HF registry 2017	8505	18398	1416	3285	74.1%	1.13 [1.05, 1.22]
Song 2020	48	215	15	80	1.0%	1.25 [0.65, 2.38]
Xu 2020	62	202	18	94	1.0%	1.87 [1.03, 3.39]
**Subtotal (95% CI)**		**25370**		**5285**	**100.0%**	**1.23 [1.15, 1.31]**
Total events	10454		1822			
Heterogeneity: Chi² = 28.49, df = 4 (P < 0.00001); I² = 86%						
Test for overall effect: Z = 6.30 (P < 0.00001)						
**6.2.2 Composite of ACM and HF hospitalization**						
Farre 2017	1277	2232	272	504	5.4%	1.14 [0.94, 1.38]
Gu 2018	311	481	73	131	1.2%	1.45 [0.98, 2.15]
KorAHF registry 2020	2532	3182	703	875	6.4%	0.95 [0.79, 1.15]
Lam 2018	522	1209	103	256	2.7%	1.13 [0.86, 1.49]
Song 2020	84	215	23	80	0.6%	1.59 [0.91, 2.77]
SwedeHF registry 2017	13006	22954	4551	8897	80.6%	1.25 [1.19, 1.31]
Vicent 2019	253	583	109	227	2.5%	0.83 [0.61, 1.13]
Xu 2020	73	202	25	94	0.6%	1.56 [0.91, 2.68]
**Subtotal (95% CI)**		**31058**		**11064**	**100.0%**	**1.22 [1.16, 1.27]**
Total events	18058		5859			
Heterogeneity: Chi² = 16.72, df = 7 (P = 0.02); I² = 58%						
Test for overall effect: Z = 8.65 (P < 0.00001)						

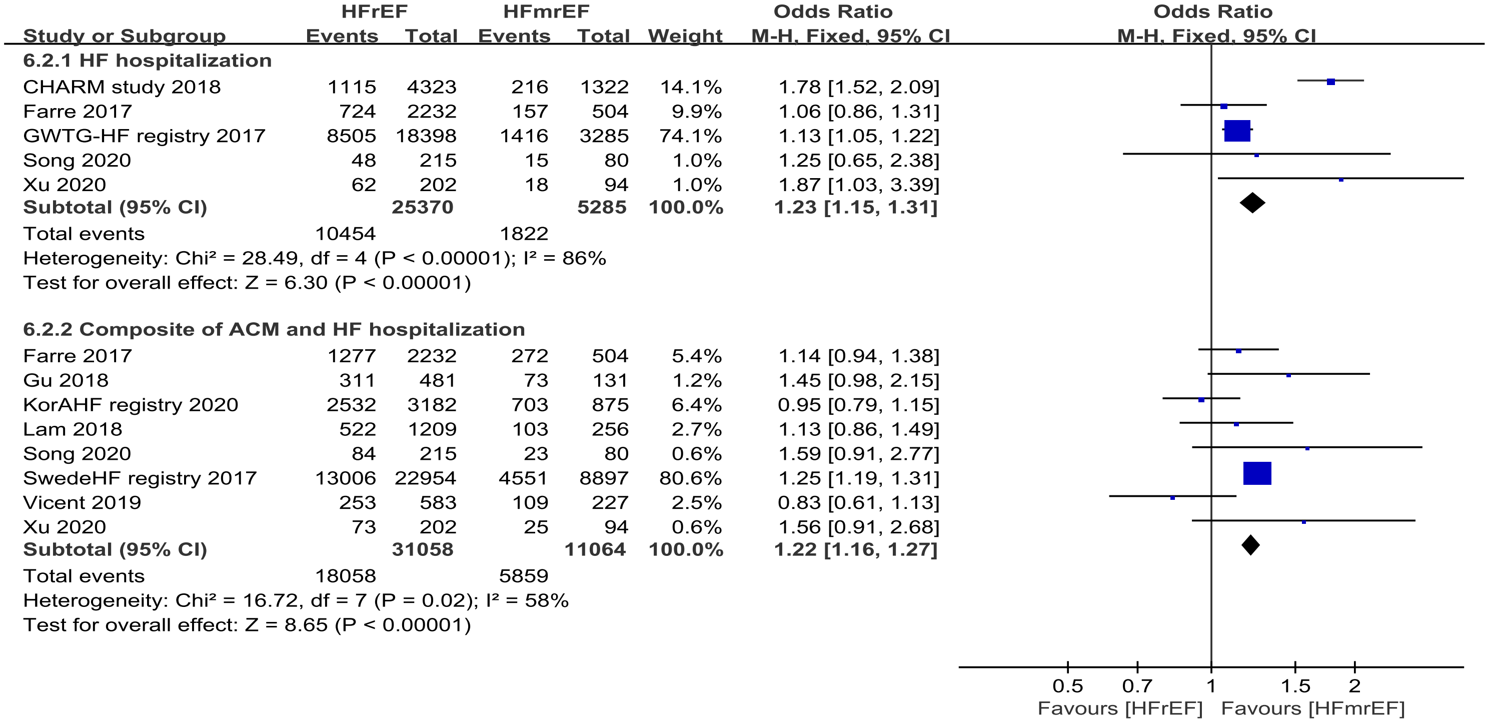

Study or Subgroup	HFmrEF Events	HFmrEF Total	HFpEF Events	HFpEF Total	Weight	Odds Ratio M-H, Fixed, 95% CI
**6.3.1 HF hospitalization**						
CHARM study 2018	216	1322	343	1953	13.8%	0.92 [0.76, 1.10]
Farre 2017	157	504	378	844	11.6%	0.56 [0.44, 0.70]
GWTG-HF registry 2017	1416	3285	7072	18299	73.1%	1.20 [1.12, 1.30]
Song 2020	15	80	19	110	0.8%	1.11 [0.52, 2.34]
Xu 2020	18	94	16	109	0.7%	1.38 [0.66, 2.88]
**Subtotal (95% CI)**		**5285**		**21315**	**100.0%**	**1.09 [1.02, 1.16]**
Total events	1822		7828			
Heterogeneity: Chi² = 42.24, df = 4 (P < 0.00001); I² = 91%						
Test for overall effect: Z = 2.52 (P = 0.01)						
**6.3.2 Composite of ACM and HF hospitalization**						
Farre 2017	272	504	564	844	6.2%	0.58 [0.46, 0.73]
Gu 2018	73	131	161	290	1.4%	1.01 [0.67, 1.53]
KorAHF registry 2020	703	875	1088	1357	5.4%	1.01 [0.82, 1.25]
Lam 2018	103	256	199	574	2.4%	1.27 [0.94, 1.72]
Song 2020	23	80	32	110	0.6%	0.98 [0.52, 1.86]
SwedeHF registry 2017	4551	8897	5375	9595	81.3%	0.82 [0.78, 0.87]
Vicent 2019	109	227	255	610	2.3%	1.29 [0.95, 1.75]
Xu 2020	25	94	17	109	0.4%	1.96 [0.98, 3.91]
**Subtotal (95% CI)**		**11064**		**13489**	**100.0%**	**0.85 [0.80, 0.89]**
Total events	5859		7691			
Heterogeneity: Chi² = 34.73, df = 7 (P < 0.0001); I² = 80%						
Test for overall effect: Z = 6.31 (P < 0.00001)						

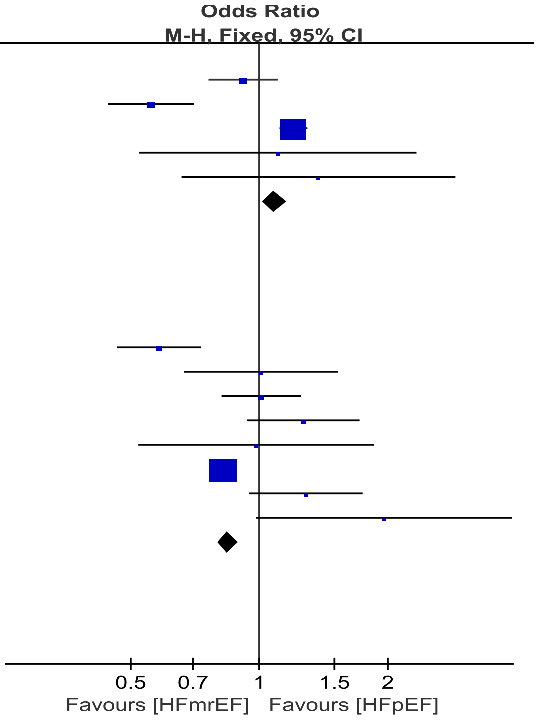

eFigure 4. Forest plot of the odds ratio (OR) and 95% confidence interval (CI) for other endpoints among three categories HF.
